# Identification of acrolein metabolites in human buccal cells, blood, and urine after consumption of commercial fried food

**DOI:** 10.1002/fsn3.1001

**Published:** 2019-04-01

**Authors:** Tse‐Wen Wang, Jin‐Hui Liu, Han‐Hsing Tsou, Tsung‐Yun Liu, Hsiang‐Tsui Wang

**Affiliations:** ^1^ Institute of Food Safety and Health Risk Assessment National Yang‐Ming University Taipei Taiwan; ^2^ Institute of Environmental and Occupational Health Sciences National Yang‐Ming University Taipei Taiwan; ^3^ Department of Pharmacology National Yang‐Ming University Taipei Taiwan

**Keywords:** 3‐HPMA, Acr‐dG adducts, acrolein, buccal cells, fried food

## Abstract

**Scope:**

Acrolein is a highly electrophilic α,β‐unsaturated aldehyde and is associated with human diseases. It is formed by Maillard reaction during food processing and could be detected in the emissions of overheated cooking oils. Consequently, humans are at risk of acrolein exposure through consumption of such prepared food.

**Methods and results:**

We conducted three human studies that healthy subjects (21–30 years) were served fried foods including fried chicken and French fries from three commercial fast food restaurants. Acrolein‐related metabolites including urinary 3‐hydroxypropyl mercapturic acid (3‐HPMA), serum acrolein‐protein conjugates (Acr‐FDP), and buccal acrolein‐induced DNA damages (Acr‐dG adducts) along with GSH levels in serum or buccal cells were investigated for different times after consumption.

**Conclusion:**

Urinary 3‐HPMA levels were increased after 2‐hr consumption of fried food with an elimination half‐life of 10 hr. In addition, increased Acr‐dG adducts in oral cavity were inversely correlated to buccal glutathione (GSH) levels after consumption. However, there was no significant change in systemic GSH levels or Acr‐FDP adducts in serum. These results indicate that exposure of acrolein from consuming fried food affects local oral cavity homeostasis. This may provide a possible link between intake of fried food and increased risk of upper aerodigestive tract cancers.

## 1 INTRODUCTION

Acrolein (2‐propenal), the most reactive α, β‐unsaturated aldehydes, is a highly mutagenic and highly oxidizing environmental toxin. It is associated with a multitude of human diseases, including cardiovascular diseases, diabetes mellitus, Alzheimer's diseases, and stroke (Moghe et al., 2015). The most well‐studied source of acrolein exposure is through tobacco smoking, which has been shown to be associated with asthma, COPD, and lung cancer (Bein & Leikauf, 2011). However, its dietary exposure and consequences are unclear. Acrolein is formed from amino acids, fats, or carbohydrates during thermal food processing (Stadler & Lineback, 2008). During the preparation of carbohydrate‐containing food, acrolein can be formed in the course of the Maillard reaction (Alarcon, 1976; Ferretti & Flanagan, 1971). Also, acrolein could be detected in the emissions of varieties of heated or overheated cooking oils, and as such is found abundantly in fried foods such as French fries (Osorio & Lourdes Cardeal, 2011). Consequently, humans are at risk of acrolein exposure through consumption of such prepared food (Abraham et al., 2011; Stevens & Maier, 2008). Oral ingestion of acrolein has been shown to exacerbate myocardial damage during ischemic heart attack and is associated with nephrotoxicity, hepatotoxicity, and reduced life span (Irwin, 2006; Parent, Caravello, Balmer, Shellenberger, & Long, 1992; Srivastava et al., 2011; Wang et al., 2008). Therefore, acrolein exposure is an important, yet underestimated food safety issue.

Previous studies have shown that human biomonitoring of the main metabolites of acrolein in urine is a better estimate of food exposure due to analytical problems with current methods for acrolein determination in food (Abraham et al., 2011). The main route of acrolein elimination involves the production of 3‐hydroxypropyl mercapturic acid (3‐HPMA) by conjunction with glutathione (GSH), the primary metabolite of acrolein found in urine (Stevens & Maier, 2008), which has been used as a biomarker for Acr exposure (Carmella et al., 2007). The biological effects of acrolein are a consequence of its reactivity with SH groups and biological nucleophiles such as guanine in DNA to form mutagenic α‐ and γ‐hydroxy‐1,N^2^‐cyclic propano‐2′‐deoxyguanosine (α‐OH‐Acr‐dG and γ‐OH‐Acr‐dG) adducts in human cells (Kanuri et al., 2002; Minko et al., 2003; Sanchez et al., 2003; Tang et al., 2011; Wang, 2012; Wang, Zhang, Hu, & Tang, 2009; Yang et al., 2003). In addition, acrolein can react with cysteine, lysine, histidine, and arginine residues in critical regions of nuclear factors, proteases, and other proteins (Cai, Bhatnagar, & Pierce, 2009; Kehrer & Biswal, 2000; LoPachin, Gavin, Petersen, & Barber, 2009) to generate acrolein‐conjugated protein (Acr‐FDP). Acr‐PC levels have been shown to correlate with neuronal damage of Alzheimer's disease patients (Bradley, Markesbery, & Lovell, 2010; Calingasan, Uchida, & Gibson, 1999; Dang, Arseneault, Murthy, & Ramassamy, 2010) and stroke patients (Igarashi & Kashiwagi, 2011; Saiki et al., 2009; Tomitori et al., 2005; Yoshida et al., 2010,2011). Therefore, the mechanisms of acrolein toxicity include formation of proteins/DNA adducts, depletion of GSH, and interference with cell signaling pathways such as oxidative stress signaling, nuclear factor‐kB, and other nuclear factors (Lambert et al., 2007,2005; Tirumalai, Rajesh Kumar, Mai, & Biswal, 2002; Wu et al., 2006). Since oral cavity tissues are the first to encounter food, their responses to harmful stimuli are critical in maintaining local homeostasis. Hence, considering the highest level of acrolein exposure through food substances and its potential chronic toxicity, buccal cells could be a good surrogate to investigate acrolein exposure from consuming fried food.

It has been shown that human biomonitoring of an acrolein urinary metabolite allows rough estimates of acrolein exposure in the range of a few μg/kg body weight/day (Watzek et al., 2012). High acrolein exposure could be ten times higher after the consumption of certain foods, such as fried potato chips (Abraham et al., 2011; Watzek et al., 2012). However, the connection of the dietary acrolein exposure and health risk is uncertain. In this study, we conducted three human studies that 21–30 aged healthy volunteers were recruited and served fried foods including fried chicken and French fries from three commercial fast food restaurants. Acrolein‐related metabolites including urinary 3‐HPMA, serum Acr‐FDP, and buccal acrolein‐induced DNA damages (Acr‐dG adducts) along with GSH levels in serum or buccal cells were investigated for different times after consumption of fried food. This study may help understand acrolein effect on local or systemic homeostasis and its possible impact on human health through consumption of fried food.

## 2 MATERIALS AND METHODS

### 2.1 Subjects and collection of buccal cells, blood, and urine

A total of 19 healthy subjects aged 21–30 were recruited for participation in the study. All participants gave informed consent for participation and were interviewed by a well‐trained interviewer. Our study protocol was approved by the Institutional Review Board of Taipei Veterans General Hospital (IRB# 2017‐11‐003BC). Experiments were conducted in accordance with the Declaration of Helsinki principles. Buccal cells, urine samples, and blood samples were collected after the interviews. The questionnaire used in the interview sought detailed information on cigarette smoking and alcohol consumption, occupational history, family disease, dietary history, and general demographic data. For the collection of buccal cells, subjects were asked to rinse their mouths with water, followed by rinsing with antiseptic mouthwash and a subsequent water rinse. A trained technician placed one buccal brush against the inside of the cheek and scraped the blush against the center of a subject's cheek, applying firm pressure throughout the process. The brush was moved up and down as well as back and forth to ensure maximum cell collection, and the inside of the mouth was scraped at least 10–15 times. The procedure was repeated to obtain a second buccal swab sample on the other side of the cheek. A total of two buccal swabs were collected for each subject. Buccal cells were stored at −30°C until use. Blood containing 3 U/ml heparin was centrifuged at 1,500 ***g*** for 10 min at 4°C, and plasma was carefully collected to avoid contamination by erythrocytes. Plasma and urine samples were kept at −80°C until use.

### 2.2 Detection of 3‐hydroxypropyl mercapturic acid (3‐HPMA) and creatinine in urine

Analysis of 3‐HPMA and creatinine in urine was based on methods previously described (Tsou et al., 2018). For analysis of 3‐HPMA in urine, solid phase extraction with Isolute ENV + cartridges (Biotage, Charlotte, NC, USA) was used to prepare each sample before liquid chromatography–tandem mass spectrometry (LC/MS/MS) analysis. High‐performance liquid chromatography (HPLC) was performed using an Agilent 1100 Series HPLC system with a quaternary pump (Agilent G1311A), a vacuum degasser (Agilent G1322A), and an autosampler (Agilent G1313A). The HPLC was directly connected to a triple quadrupole mass spectrometer (Finnigan TSQ Quantum Discovery MAX, Thermo Electron Corporation, Netherlands) equipped with an electrospray ionization (ESI) interface and a 10‐port valve. A Hypersil GOLD aQ C18 2.1 mm × 150 mm, 3 μm column (Thermo Fisher Scientific), was used for LC separation. For analysis of creatinine, HPLC was performed using an Agilent Series 1260 HPLC system with a binary pump (Agilent G1312B), a vacuum degasser (Agilent G1322A), and an autosampler (Agilent G1367E). The HPLC was directly connected to a variable wavelength detector (Agilent G1314F). A NUCLEODUR C18 HTec 250 mm × 4.6 mm × 4.6 μm column was used for LC separation. The samples were analyzed at a wavelength of 240 nm via a variable wavelength detector.

### 2.3 Slot blot assay for Acr‐dG and 8‐oxo‐dG detection

Analysis of Acr‐dG adducts or 8‐oxo‐dG adducts in DNA samples was based on previously described methods (Lee et al., 2014; Wang et al., 2013). Control genomic DNA was modified with Acr (0.5, 1, 2, and 5 mM) at 37°C for 24 hr and purified with repeated phenol/ether extraction as Acr‐dG adduct standards. For 8‐oxo‐dG adduct standard, control genomic DNA was modified with H_2_O_2_ (1, 2, 5, and 10 mM) at 37°C for 1 hr. After purification, modified DNA was precipitated with ethanol and dissolved in TE buffer (pH 8.0). Buccal DNA was extracted from buccal cells using Puregene buccal cell *core *kits *(*Qiagen, *Valencia, CA) *according *to the *manufacturer's instructions. Modified DNA or buccal DNA (0.25 μg) was loaded onto PVDF membranes using a Bio‐Dot SF microfiltration apparatus (Bio‐Rad, Hercules, CA). WesternDot™ 625 Western Blotting Kits (Invitrogen) were used for Western blot analysis according to the manufacturer's instructions. After blocking for 1 hr at room temperature in blocking buffer, the membrane was probed overnight at 4°C with anti‐Acr‐dG mouse monoclonal antibodies (Pan et al., 2012) or anti‐8‐oxo‐dG mouse monoclonal antibody (Abcam, Ab62623). After washing with washing buffer to remove unbound primary antibodies, quantum dot‐conjugated secondary antibodies (1:1,000 dilution) were added for 2 hr at room temperature. Ultimately, Acr‐dG adducts were detected using a UVP BioDoc‐It™ imaging system, and band density was quantified with UVP imaging software. After antibody detection, the same membrane was stained with methylene blue (Molecular Research Center, Cincinnati, OH) to indicate the amount of DNA.

### 2.4 Slot blot assay for acrolein‐protein detection

Analysis of acrolein‐protein conjugates (Acr‐FDP) using slot blot analysis was based on methods previously described (Tsou et al., 2018). Briefly, for whole cell lysate extraction, cells will be washed in PBS and lysed by RIPA buffer with 1% of protease inhibitor cocktail (Calbiochem). Protein levels in whole cell lysate or serum were measured by BCA Protein Assay Kit (Pierce) using bovine serum albumin (BSA) as a standard. Acrolein‐modified BSA, whole cell lysate, or plasma samples (10 μg) were loaded onto PVDF membranes using a Bio‐Dot SF microfiltration apparatus (Bio‐Rad, Hercules, CA). WesternDot™ 625 Western Blot Kits (Invitrogen) were used for blot analysis according to the manufacturer's instructions. Ultimately, Acr‐FDP adducts were detected using UVP BioDoc‐It™ Imaging System and band density was quantified with UVP imaging software. After antibody detection, the membrane was stained with Ponceau S (Sigma) to provide an estimated amount of protein present.

### 2.5 Glutathione (GSH) assay

The level of total glutathione (GSH + GSSG) was determined with a Glutathione Assay Kit (Sigma) following the manufacturer's protocol. Briefly, a solution of 5% 5‐sulfosalicylic acid was added to plasma or buccal cells to deproteinize the samples. Glutathione was measured in a kinetic assay based on the reaction of 5, 5‐dithiobis (2‐nitrobenzoic acid) (DTNB) to yellow TNB, which was spectrophotometrically measured at 412 nm. The amount of total glutathione was determined with a standard cure of reduced glutathione.

### 2.6 Statistical analyses

Descriptive statistics were presented as the mean ± *SD* or as the number (percentage). Shapiro–Wilk test was used to test for normality. Student's *t* tests were used to determine statistical significance. Pearson correlation analysis was used to analyze the correlation between acrolein‐related metabolites and clinical parameters. A minimum of three independent replicate experiments of slot blot analysis or isotope dilution HPLC mass spectrometry were performed to justify the use of the statistical tests. All calculated *p*‐values were two‐tailed. Statistical significance was defined as a *p* < 0.05. All analyses were performed with the IBM SPSS Statistics software package, version 23.0.

## 3 RESULTS

### 3.1 Subjects and collection of buccal cells, blood, and urine

A total of 19 healthy subjects aged 21–30 were recruited and served fried foods including fried chicken and French fries from three commercial fast food restaurants (Study 1: N‐restaurant, Study 2: P‐restaurant, and Study 3: K‐restaurant). The study was approved by the Taipei Veterans General Hospital Institutional Review Board. All subjects provided informed consent in their native language to participate in the study. After consumption of fried food, these subjects’ buccal cells, blood, and urine were collected for different times (0–24 hr). Designed questionnaires regarding personal health history, smoking history, drinking history, and dietary intake on that day will be asked to fill out at the same time as their buccal cells, blood, and urine collected. Baseline sociodemographic variables of healthy subjects in three studies are shown in Table 1.

**Table 1:** Baseline sociodemographic variables of healthy subjects in three studies

	Study 1	Study 2	Study 3
*N*	19	9	9
Female (%)	63.2^ns^	77.8^ns^	66.7^ns^
Male/female	7/12	2/7	3/6
Age (years)	23.7 ± 0.7^ns^	23.6 ± 0.53^ns^	24.22 ± 3.21^ns^
Range	24–25	23–24	21–30
Smoker	0	0	0
Amount of intake of fried food			
Mean ± *SD* (g/meal)	317.2 ± 106.0^ns^	339.8 ± 159.2^ns^	356.8 ± 173.2^ns^
Fast food restaurant	*N*‐restaurant	P‐restaurant	K‐restaurant

Note

The values are shown in mean ± *SD*.

ns, p ≧0.05 between different studies by Student's *t* test.

### 3.2 Increased urinary 3‐HPMA in healthy subjects after consuming fried food from three commercial restaurants

**Figure 1:**
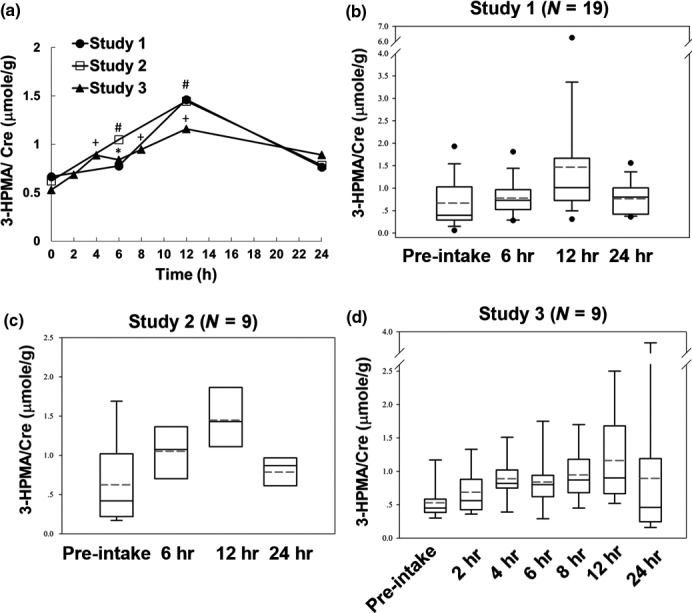
Levels of 3‐HPMA/Cre in urine of control subjects after consuming one meal of fried food at different times. (A) Average urinary 3‐HPMA/Cre levels of control subjects at different times from three studies were measured using isotope dilution HPLC mass spectrometry. Student's *t* tests were used to determine statistical significance, and two‐tailed *p*‐values are shown. *^, #, +^ *p* < 0.05 between pre‐intake (0 hr) and 2–24 hr after intake in Study 1, Study 2, and Study 3, respectively. (B‐D) Box‐and‐whisker plot of 3‐HPMA/Cre levels in control subjects at different time points from three studies

3‐HPMA, an acrolein‐GSH conjugate, is a metabolite formed by detoxification of acrolein (Stevens & Maier, 2008). Here, we established a sensitive methodology to analyze the levels of urinary 3‐HPMA using isotope dilution HPLC mass spectrometry (Tsou et al., 2018) and examined the urine of healthy subjects after consumption of fried food. Kinetic parameters and amounts of 3‐HPMA excreted in urine within 24 hr after intake of fried food from three commercial restaurants are shown in Table 2 and Figure 1. Similar kinetic pattern was observed in these three studies. At baseline (pre‐intake) of these three studies, the median 3‐HPMA concentration was 0.53–0.67 μmole/g creatinine. After intake of the fried food, an increase in 3‐HPMA excretion was observed in the first sampling period (0–2 hr), reaching Cmax at 12 hr. Thereafter, concentrations decreased, approaching almost baseline level at 24 hr. The apparent terminal elimination half‐life for 3‐HPMA was 10 hr (Table 3).

**Table 2:** Urinary 3‐HPMA levels in three human studies after consumption of fried food from three commercial restaurants

	Creatinine mean ± *SD* (mg/dl)	3‐HPMA mean ± *SD* (ng/ml)	3‐HPMA/Cre. mean ± *SD* (µmole/g)
Study 1			
Before intake	150.94 ± 118.57	185.65 ± 172.25	0.67 ± 0.52
After intake 6 hr	80.77 ± 47.29**	114.69 ± 67.98	0.78 ± 0.38
After intake 12 hr	61.96 ± 44.84***	154.75 ± 114.85	1.47 ± 1.38^a^
After intake 24 hr	127.92 ± 78.40	194.69 ± 136.00	0.77 ± 0.36
Study 2			
Before intake	147.59 ± 107.42	160.97 ± 124.53	0.63 ± 0.57
After intake 6 hr	120.00 ± 69.51	304.18 ± 245.27	1.05 ± 0.44^a^
After intake 12 hr	89.14 ± 35.24	277.31 ± 124.56	1.45 ± 0.54^a^
After intake 24 hr	147.52 ± 60.36	232.87 ± 86.84	0.79 ± 0.26
Study 3			
Before intake	98.93 ± 54.73	114.47 ± 71.57	0.53 ± 0.26
After intake 2 hr	120.12 ± 60.54	191.34 ± 160.30	0.69 ± 0.32
After intake 4 hr	123.26 ± 53.13	249.12 ± 160.52^a^	0.89 ± 0.30^a^
After intake 6 hr	76.11 ± 43.66	147.83 ± 110.99	0.84 ± 0.40
After intake 8 hr	123.54 ± 44.67	263.79 ± 143.98***	0.95 ± 0.37^a^
After intake 12 hr	93.65 ± 50.33	244.93 ± 217.97	1.16 ± 0.66^a^
After intake 24 hr	138.80 ± 54.05	216.34 ± 162.24	0.89 ± 1.08

* *p* < 0.05, **p < 0.01, **p < 0.005, Student's *t* tests were used to determine statistical significance between before intake and after intake, and two‐tailed *p*‐values are shown.

**Table 3:** Kinetic parameters of 3‐HPMA levels in urine samples of three human studies after consumption of fried food from three commercial restaurants. (3‐HPMA/Cre levels are expressed as μmole/g creatinine, mean ± *SD*)

	Study 1	Study 2	Study 3
*C*_predose_	0.67 ± 0.52	0.63 ± 0.57	0.53 ± 0.26
*C*_max_	1.47 ± 1.38	1.45 ± 0.54	1.16 ± 0.66
*t*_1/2_	10 hr	9.4 hr	10.7
*t*_max_	12 hr	12 hr	12 hr
AUC	22.8	24.2	21.5

Note

AUC: area under curve, μmole/ g*h.

### 3.3 Decrease in buccal GSH level, but not in serum GSH level after consuming fried food

**Figure 2:**
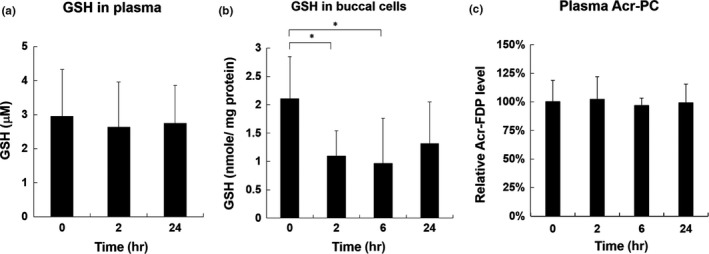
Total GSH levels in plasma and buccal cells and Acr‐FDP levels in plasma of control subjects after consuming one meal of fried food at different times. Total GSH levels in plasma (A) or buccal cells (B) of Study 3 were analyzed using Glutathione Assay Kit (Sigma). (C) Acr‐FDP levels in plasma of Study 3 were analyzed using slot blot analysis as described in Materials and Methods. Bar graphs of data collected from two independent experiments. Data were presented as the mean ± *SD*. Student's *t* tests were used to determine statistical significance, and two‐tailed *p*‐values are shown. *p < 0.05

Since GSH is a major cellular scavenger for acrolein, the level of GSH affects acrolein‐induced cellular toxicity (Stevens & Maier, 2008; Wang et al., 2012). We found significant decrease in buccal GSH level after 2‐hr‐consuming fried food, while there was no change in plasma GSH levels after 2‐hr‐ or 24‐hr‐consuming fried food (Figure 2a‐b). In addition, buccal GSH level was lower than basal level after 24‐hr‐consuming fried food, even though it did not reach statistical significance.

### 3.4 Slightly increased acrolein‐modified protein (Acr‐PC) adducts in the plasma of healthy subjects after consuming fried food

We examined the plasma samples collected from healthy subjects after consumption of fried food by slot blot analysis using anti‐Acr‐FDP antibody to analyze acrolein‐modified protein conjugates, namely *N*ε‐(3‐formyl‐3,4‐dehydropiperidino)lysine (Acr‐FDP) (Uchida et al., 1998). Using slot blot analysis, Acr‐FDP levels in plasma were slightly but not significantly increased after 2‐hr‐, 6‐hr‐, or 24‐hr‐consuming fried food (Figure 2c).

### 3.5 Increased Acr‐dG adducts and 8‐oxo‐dG adducts in buccal DNA of healthy subjects after consuming fried food

**Figure 3:**
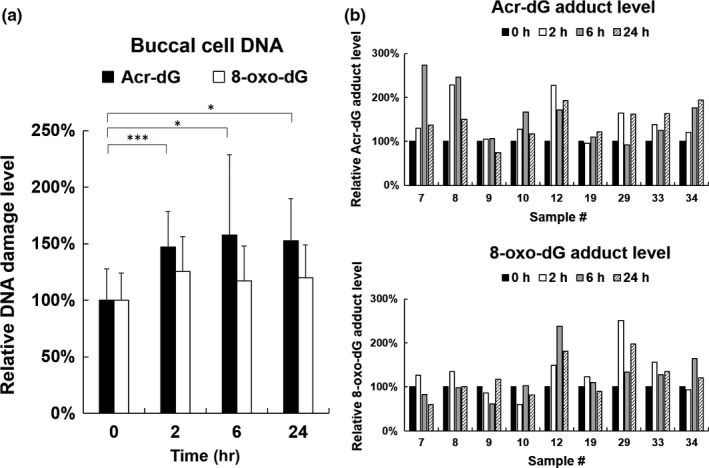
Slot blot analysis of Acr‐dG and 8‐oxo‐dG adducts in buccal DNA of control subjects after consuming one meal of fried food at different times. (A) Relative Acr‐dG adduct or 8‐oxo‐dG adduct levels in buccal cells of Study 3 (*N* = 9). Bar graphs of data collected from two independent slot blot experiments. Data were presented as the mean ± *SD*. Student's *t* tests were used to determine statistical significance, and two‐tailed *p*‐values are shown. *p < 0.05, ***p < 0.005. (B) Relative Acr‐dG adduct level (upper panel) and 8‐oxo‐dG adduct level (lower panel) in buccal cells of individual control subjects (*N* = 9) from Study 3

We examined buccal DNA samples collected from healthy subjects after consumption of fried food by slot blot analysis using anti‐Acr‐dG antibodies to analyze acrolein‐induced DNA adducts (α‐ and γ‐hydroxy‐1,N^2^‐cyclic propano‐2′‐deoxyguanosine (α‐OH‐Acr‐dG and γ‐OH‐Acr‐dG) adducts) that have been previously described (Lee et al., 2014; Wang et al., 2013). We found that the Acr‐dG levels in the buccal DNA of healthy subjects were 1.5‐fold higher after 2‐hr‐consuming fried food (*p* < 0.001) and Acr‐dG adduct levels were not decreased until 24 hr (Figure 3). Since acrolein has been shown to induce oxidative stress resulting in oxidative DNA damages (Li, Jiang, Geng, Cao, & Zhong, 2008; Wang, Chen, Weng, Yang, & Tang, 2016), we also investigate the level of 8‐oxo‐deoxyguanosine (8‐oxo‐dG), a biomarker of oxidative DNA damages in buccal DNA in these healthy subjects after consuming fried food. Results in Figure 3 showed that 8‐oxo‐dG levels in buccal DNA of healthy subjects were 1.2‐fold higher after 2‐hr‐consuming fried food (*p* = 0.077). These results suggest that consumption of fried food increased Acr‐dG adducts as well as oxidative DNA damages in oral cavity. However, the increase in Acr‐dG adduct was higher than 8‐oxo‐dG adducts after consumption of fried food.

## 4 DISCUSSION

Acrolein can be formed from carbohydrates, vegetable oils and animal fats, and amino acids during preparation of food (Stadler & Lineback, 2008). Also, acrolein could be detected in the emissions of varieties of heated or overheated cooking oils, and as such is found abundantly in fried foods such as French fries (Osorio & Lourdes Cardeal, 2011). Consequently, humans are at risk of acrolein exposure through consumption of food (Abraham et al., 2011; Stevens & Maier, 2008). However, its dietary exposure and consequences are underinvestigated. Here, we performed three studies to analyze acrolein exposure in healthy subjects after consumption of fried food from three commercial fast food restaurants. Results in Figure 1a showed that urinary 3‐HPMA concentrations increased within the first 2 hr after test meal intake, reaching Cmax at 12 hr. Thereafter, concentrations decreased, approaching almost baseline level at 24 hr. The apparent terminal elimination half‐life for 3‐HPMA was 10 hr (Table 3). The kinetic parameters of acrolein metabolism are similar in these three studies. Previous studies have shown that the uptake of commercial potato crisps led to an increase in 3‐HPMA, with Cmax reached at 2–4 hr, with a decrease close to baseline and an elimination half‐life of 12 hr (Watzek et al., 2012). Different times of reaching Cmax are probably due to different absorption rates of acrolein between different types of fried food.

Intracellular GSH is a major scavenger for acrolein, and previous studies have shown that cellular GSH levels are inversely correlated with acrolein‐induced cytotoxicity (Wang et al., 2012). Here, we found that systemic GSH level or tissue GSH levels responded differently after intake of fried food. Oral cavity tissues are the first to encounter food, and their responses to harmful stimuli are critical in maintaining local homeostasis. Results showed that buccal GSH levels were significantly decreased after 2‐hr‐ and 6‐hr‐consuming fried food (Figure 2b). After 24‐hr‐consuming fried food, buccal GSH levels were lower than basal level, but not statistically significant. On the other hand, there was no change in serum GSH levels (Figure 2a). These results indicate that exposure of acrolein through intake of fried food affects local homeostasis in oral cavity tissues more than systemic homeostasis. The possible explanation is that the abundance of systemic GSH level is enough to detoxify acrolein from one meal of fried food. On the other hand, buccal GSH levels in oral cavity may be depleted by acrolein within a short period of time after consuming fried food, resulting in local cellular toxicity. Indeed, we found that acrolein‐induced DNA damages (Acr‐dG or 8‐oxo‐dG levels) increased in buccal cells after 2‐hr‐, 6‐hr‐, and 24‐hr‐consuming fried food (Figure 3), but no significant increase in acrolein‐induced protein conjugates (Acr‐FDP adducts) in serum (Figure 2c). These results are consistent with animal studies showing that target organs of toxicity of acrolein are primarily the local tissues affected (Auerbach, Mahler, Travlos, & Irwin, 2008; Faroon et al., 2008). The authors found that oral exposure of acrolein leads to gastrointestinal symptoms, gastric ulcers, and/or gastric bleeding, whereby the severity is dose‐dependent. Therefore, the effect of acrolein on systemic homeostasis after long‐term consumption of fried food needs further investigation.

Previous studies have suggested that a high level of oral exposure to Acr through consumption of food or other sources induces detrimental effects on the oral cavity, including salivary quality and contents, oral resistance to oxidative stress, and stress mechanism activation in a variety of oral cells (Aizenbud, Aizenbud, Reznick, & Avezov, 2016). Here, we found that acrolein‐induced DNA adducts, that is Acr‐dG adducts, were significantly increased in buccal cells after 2‐hr and 6‐hr‐consuming fried food (Figure 3) and the levels were not decreased after 24‐hr‐consuming fried food, indicating that Acr‐dG adducts were not repaired. Our previous studies have shown that Acr inhibits DNA repair and Acr‐dG adducts are mutagenic (Kanuri et al., 2002; Minko et al., 2003; Sanchez et al., 2003; Tang et al., 2011; Wang, 2012; Wang et al., 2009; Yang et al., 2003). Therefore, it is reasonable to propose that long‐term consumption of fried food may cause accumulation of these DNA damages and formation of DNA mutations, finally resulting in cancer formation in oral cavity or upper aerodigestive tract. Previous studies have shown that a diet rich in fried food was related to a moderate increase in risk of upper aerodigestive tract cancers in men (Galeone et al., 2005). This result probably provides a possible mechanism linking intake of fried food and increased risk of upper aerodigestive tract cancers. However, the mutagenicity or carcinogenicity of acrolein after oral exposure should be examined and clarified meticulously in order to make the conclusion.

There are some limitations in this study. One is that oral intake of acrolein can be overestimated from urinary metabolite concentrations because both endogenous formation and inhalation exposure (in particular kitchen vapors, road traffic, passive smoking) could be relevant. Numerous studies have reported that tobacco smoke has high acrolein contents and smokers have a significantly higher acrolein exposure than nonsmokers (Alwis, deCastro, Morrow, & Blount, 2015; Carmella et al., 2007; DeJarnett et al., 2014; Kassem et al., 2018; Stevens & Maier, 2008). Furthermore, smoking of tobacco products is equivalent to or exceeds the total human exposure to Acr from all other sources (Gerberding, 2007). Although the participants recruited in this study are nonsmokers, we still could not exclude other exposure sources other than active smoking or intake of food. Another limitation is that we were unable to restrictedly control kinds and amounts of food in other meals these participants consumed before or after this designed meal during our urine samplings (2–20 hr). Even though these uncertainties, we still can see increase in urinary 3‐HPMA or Acr‐induced DNA damages after 2‐hr‐consuming fried food (Figures 1 and 3). The other limitation is missing data on detection of acrolein on fried food in these three commercial fast food restaurants. Previous studies have shown that there are no available validated analytical techniques for the detection of acrolein in food; hence, a valid estimate of the oral acrolein exposure from content and consumption data is currently not feasible (Abraham et al., 2011). Therefore, the development of valid analytical methods for food as well as the examination of a broad range of food in ready‐to‐eat form is our current task.

In this present study, we presented that acrolein‐related metabolites including urinary 3‐HPMA and buccal acrolein‐induced DNA adducts were increased after 2‐hr consumption of fried food from three different commercial fast food restaurants. In addition, buccal GSH levels, but not plasma GSH levels, were inversely correlated to acrolein‐induced toxicity in oral cavity after consumption of fried food. These results indicate that exposure of acrolein from consuming one meal of fried food affects local oral cavity homeostasis. Therefore, the effect of acrolein on systemic homeostasis after long‐term consumption of fried food needs further investigation. This may provide a possible link between intake of fried food and increased risk of upper aerodigestive tract cancers.

## CONFLICTS OF INTEREST

The authors have no actual or potential conflicts of interest.

## AUTHOR CONTRIBUTIONS

T‐W. W., J‐H L., H‐H T., and H‐T W. performed experiments; T‐Y L. and H‐T W. designed experiments and participated in manuscript writing.

## ETHICAL APPROVAL

All participants gave informed consent for participation and were interviewed by a well‐trained interviewer. Our study protocol was approved by the Institutional Review Board of Taipei Veterans General Hospital (IRB# 2017‐11‐003BC). Experiments were conducted in accordance with the Declaration of Helsinki principles.
